# The pretherapeutic systemic inflammation score is a prognostic predictor for elderly patients with oesophageal cancer: a case control study

**DOI:** 10.1186/s12885-023-10982-4

**Published:** 2023-06-04

**Authors:** Chunyue Huang, Mengyao Wang, Liwen Chen, Hongmei Wang, Donglan Huang, Jianjun Shi, Weijun Zhang, Yunhong Tian, Yujia Zhu

**Affiliations:** 1 Department of Radiation Oncology, Affiliated Cancer Hospital & Institute of Guangzhou Medical University, Guangzhou, China; 2 Department of Medical Imaging, Affiliated Cancer Hospital & Institute of Guangzhou Medical University, Guangzhou, China; 3 Department of Radiation Oncology, State Key Laboratory of Oncology in Southern China, Collaborative Innovation Center for Cancer Medicine, Sun Yat-Sen University Cancer Center, Guangzhou, China

**Keywords:** Systemic inflammation score, Oesophageal carcinoma, Predictive value, Elderly patient

## Abstract

**Background:**

The systemic inflammation score (SIS), based on serum albumin (Alb) and lymphocyte-to-monocyte ratio (LMR), is a novel prognostic tool for some tumours. Studies indicate that the SIS can be used as a postoperative prognostic marker. However, its predictive value in elderly oesophageal squamous cell carcinoma (ESCC) patients treated with radiotherapy is unclear.

**Methods:**

In total, 166 elderly ESCC patients who received radiotherapy with or without chemotherapy were included. Based on different combinations of Alb and LMR levels, the SIS was divided into 3 groups, SIS = 0 (*n* = 79), SIS = 1 (*n* = 71) and SIS = 2 (*n* = 16). The Kaplan—Meier method was used for survival analysis. Univariate and multivariate analyses were performed to assess prognosis. Time-dependent receiver operating characteristic (t-ROC) curves were used to compare the prognostic accuracy of the SIS with that of Alb, LMR, neutrophil-to lymphocyte ratio (NLR), platelet-to-lymphocyte ratio (PLR), and systemic immune-inflammatory index (SII).

**Results:**

Decreased Alb and LMR were both associated with shorter OS, whereas a lower SIS was significantly associated with better outcomes. The OS of SIS = 0, SIS = 1 and SIS = 2 was 28.0 ± 2.9, 16.0 ± 2.8 and 10.0 ± 7.0 months, respectively (*p* = 0.000). Similar results were also observed for PFS. Multivariate analysis of the model with SIS revealed that the SIS was a significant independent biomarker for predicting OS and PFS. The nomogram showed that the C-index was improved to 0.677 when the SIS factor was incorporated. Furthermore, the 3-year OS rates for patients in the SIS-high group (SIS = 1 and SIS = 2) undergoing concurrent radiotherapy with a single agent (CCRT-1) and concurrent radiotherapy with two agents (CCRT-2) were 42% and 15%, respectively (*p* = 0.039). The t-ROC curve showed that the SIS was more sensitive than other prognostic factors for predicting overall survival.

**Conclusion:**

The SIS may be a useful prognostic marker in elderly patients with ESCC receiving radiotherapy alone or chemoradiotherapy. The SIS showed a better predictive ability for OS than the continuous variable Alb and could stratify patient prognosis in different therapeutic regimens. CCRT-1 may be the best treatment for SIS-high patients.

## Background

The incidence of oesophageal cancer ranks third and fourth in men and women in China, respectively, and approximately 69.8% of men with oesophageal cancer are older than 60 years of age [1]. Concurrent chemoradiotherapy using the cisplatin/5-fluorouracil regimen is the standard treatment option for patients with inoperable oesophageal cancer [2]. Although the outcomes of oesophageal cancer patients have improved during the last decade, the 5-year survival rate is less than 30% in elderly oesophageal cancer patients [3]. To identify high-risk patients and improve survival, it is essential to explore novel prognostic indicators to help oncologists make appropriate treatment decisions in advance.

Studies on the prognostic value of systemic inflammation in cancer patients have been ongoing for years [4–7]. In cancer patients, the systemic inflammatory response induces increased peripheral blood cell numbers and decreased serum albumin levels [8]. Thus, various combinations based on circulating blood cell counts have been developed to predict the outcome in various tumours. The most common predictive markers included the lymphocyte-to-monocyte ratio (LMR), neutrophil-to-lymphocyte ratio (NLR), platelet-to-lymphocyte ratio (PLR) and systemic immune-inflammatory index (SII) [9–12]. Serum albumin levels, a classic nutrition index, were also considered an inflammation related factor and reported as a prognostic marker for cancer in many studies [13–15]. These markers are routinely employed in the clinic, inexpensive to test and promising to help oncologists estimate patient outcomes. However, there are no generally accepted inflammatory scoring systems, used to predict the prognosis of cancer.

The systemic inflammation score (SIS), first created by Chang et al. [16], consists of the serum albumin (Alb) level and lymphocyte-to-monocyte ratio (LMR), and is a novel marker to assess the inflammatory and nutritional status of patients. Previous studies showed that low perioperative SIS was associated with longer postoperative survival in gastric cancer [17], oesophageal cancer [18] and breast cancer [19]. However, its survival predictive value in inoperable oesophageal cancer of elderly patients is unknown. We therefore aimed to assess the correlations between the SIS and survival outcomes in elderly patients with ESCC who received radiotherapy or chemoradiotherapy and compared the prognostic accuracy of the SIS with that of other prognostic factors (Alb, LMR, NLR, PLR, and SII).

## Methods

### Patients

We performed a retrospective analysis of 166 patients with oesophageal squamous cell cancer aged ≥ 65 years at the time of diagnosis who received radiotherapy with or without chemotherapy at Sun Yat-sen University Cancer Center or Affiliated Cancer Hospital & Institute of Guangzhou Medical University from August 2002 to February 2017. Patients were excluded based on the following criteria: (1) multiple primary oesophageal carcinoma lesions; (2) surgery before/after radiotherapy; (3) postoperative recurrence; (4) other tumours; (5) a Karnofsky performance status (KPS) < 70; (6) any concomitant infectious disease; and (7) a lack of full blood counts, serum albumin levels, and/or total cholesterol levels measured 1 month before radiotherapy. The Institutional Review Board of Sun Yat-sen University Cancer Center and Affiliated Cancer Hospital & Institute of Guangzhou Medical University approved the study protocol, which was performed according to the Declaration of Helsinki. We defined the elderly population based on social security and Medicare regulations as persons aged 65 years or older.

### Data collection

We obtained the following information from patient medical records: age, sex, Karnofsky performance status (KPS), body mass index (BMI), smoking status, tumour location, T stage, N stage, M stage, TNM stage, chemotherapy regimen, early tumour response, Charlson comorbidity index, family history of cancer, progression-free survival (PFS) and overall survival (OS). Tumour TNM stage was based on barium oesophagography, chest and abdominal computed tomography scans, and oesophageal ultrasonography when feasible. Tumours were staged according to the sixth edition of the American Joint Committee on Cancer (AJCC) staging manual.

Blood tests were performed within 2 weeks before radiotherapy. Laboratory tests included neutrophil (N), lymphocyte (L), platelet levels (P), monocyte (M), and serum albumin concentration (Alb) for the calculation of the NLR, PLR, LMR, SII and SIS indexes. NLR and PLR were defined as neutrophil or platelet counts divided by the total number of lymphocytes. The LMR was defined as the lymphocyte count divided by the monocyte count. The systemic immune-inflammatory index (SII) was calculated with the formula SII = (P × N)/L. The SIS was determined based on serum albumin level and the LMR. Owing to the lack of a widely accepted definition of SIS, in the current study, the cut-off points of Alb at 39.8 g/L and LMR at 1.68, calculated by X-tile software, were used as the cut-off for dichotomization. As shown in Table 1, patients with a serum albumin level > 39.8 g/L and LMR > 1.68 were allocated a score of 0, patients with either hypoalbuminemia (Alb ≤ 39.8 g/L) or a decrease in LMR (LMR ≤ 1.68) were allocated a score of 1, and patients with both hypoalbuminemia (Alb ≤ 39.8 g/L) and a decrease in LMR (LMR ≤ 1.68) were assigned a score of 2.

**Table 1:** Definition of the systemic inflammation score

Factor	SIS score
Serum albumin > 39.8 g/L and lymphocyte:monocyte ratio > 1.68	0
Serum albumin ≤ 39.8 g/L or lymphocyte:monocyte ratio ≤ 1.68	1
Serum albumin ≤ 39.8 g/L and lymphocyte:monocyte ratio ≤ 1.68	2

### Response and survival

The early responses were evaluated using barium oesophagography or chest and abdominal computed tomography scans at the end of treatment, and the responses were classified according to Eisenhauer’s report. Overall survival was defined as the time from admission to death or the time of analysis. Progression-free survival was calculated as the time from admission to recurrence, death from any cause or the time of analysis.

### Statistical analysis

Data were analysed using SPSS 16.0 software (SPSS Inc., Chicago, United. States). The Kaplan–Meier method was used for the survival analyses. Log-rank testing was used to compare the differences in outcomes between treatment groups. Univariate and multivariate analyses were performed using the Cox proportional hazards regression model, and all of the prognostic variables identified in the univariate analysis with *p* < 0.1 were subjected to multivariate analysis. Chi-squared tests or Fisher’s exact tests were performed to compare the different categorical variable groups. X-tile software (Yale University, New Haven, CT, USA) was used to identify the optimal cut-off values for Alb and LMR. Receiver operating characteristic (ROC) curve analysis was performed to analyse the area under the ROC curve (AUC). All tests were two-sided, and *p* < 0.05 was considered statistically significant.

## Results

### Clinical characteristics and SIS

The baseline clinicopathological characteristics of the 166 eligible patients are summarized in Table 2. Based on different combinations of serum albumin and LMR levels, the SIS was developed into 3 groups, named SIS = 0, SIS = 1 and SIS = 2 (Table 1). According to our stratification of SIS, we observed that high SIS was associated with high M stage (*p* = 0.010) and more advanced TNM stage (*p* = 0.014).

**Table 2:** The relationship between the SIS group and clinicopathological characteristics

Variables	SIS (n, n%)				*p* value
		**SIS = 0**	**SIS = 1**	**SIS = 2**	
All cases	166	79	71	16	
Age(years)					
≤ 70	72 (43.4%)	41 (51.9%)	26 (36.6%)	5 (31.2%)	0.100
> 70	94 (56.6%)	38 (48.1%)	45 (63.4%)	11 (68.8%)	
Gender					
Male	117 (70.5%)	52 (65.8%)	51 (71.8%)	14 (87.5%)	0.175
Female	49 (29.5%)	27 (34.2%)	20 (28.2%)	2 (12.5%)	
Karnofsky performance status					
< 80	13 (7.8%)	3 (3.8%)	8 (11.3%)	2 (12.5%)	0.164
≥ 80	153 (92.2%)	76 (96.2%)	63 (88.7%)	14 (87.5%)	
Body mass index(kg/m2)					
< 18.5	21 (15.9%)	9 (13.4%)	10 (20.0%)	2 (13.3%)	0.611
≥ 18.5	111 (84.1%)	58 (86.6%)	40 (80.0%)	13 (86.7%)	
Smoking status					
No	77 (46.4%)	36 (45.6%)	34 (47.9%)	7 (43.8%)	0.937
Yes	89 (53.6%)	43 (54.4%)	37 (52.1%)	9 (56.2%)	
Family history of cancer					
No	140 (84.3%)	70 (88.6%)	54 (76.1%)	16 (100%)	**0.007**
Yes	26 (15.7%)	9 (11.4%)	17 (23.9%)	0 (0%)	
Charlson Comorbidity Index					
Mean ± SD	0.60 ± 0.81	0.56 ± 0.71	0.66 ± 0.93	0.56 ± 0.73	0.716
Tumour location					
Cervical and upper thoracic	66 (39.8%)	37 (46.8%)	26 (36.6%)	3 (18.8%)	0.087
Middle and low thoracic	100 (60.2%)	42 (53.2%)	45 (63.4%)	13 (81.2%)	
T stage					
T1-2	29 (18.8%)	18(23.7%)	9(14.3%)	2 (13.3%)	0.311
T3-4	125 (81.2%)	58(76.3%)	54(85.7%)	13 (86.7%)	
M stage					
M0	101 (66.3%)	50(63.3%)	54(76.1%)	6 (37.5%)	**0.010**
M1	65 (33.7%)	29(36.7%)	17(23.9%)	10 (62.5%)	
Tumour TNM stage					
I + II	34 (21.4%)	20 (25.6%)	14 (21.5%)	0 (0%)	**0.014**
III + IV	125 (78.6%)	58 (74.4%)	51 (78.5%)	16 (100%)	
Tumour early response					
CR + PR	118 (71.1%)	59 (74.7%)	49 (69.0%)	10 (62.5%)	0.548
SD + PD	48 (28.9%)	20 (25.3%)	22 (31.0%)	6 (37.5%)	
Chemotherapy regimen					
None	63 (38.0%)	27 (34.2%)	27 (38.0%)	9 (56.2%)	0.570
Single agent	44 (26.5%)	21 (26.6%)	20 (28.2%)	3 (18.8%)	
Two agents	59 (35.5%)	31 (39.2%)	24 (33.8%)	4 (25.0%)	

The *P* value in bold indicated that statistically significant

### Associations of LMR, serum albumin and SIS with OS and PFS

**Fig. 1:**
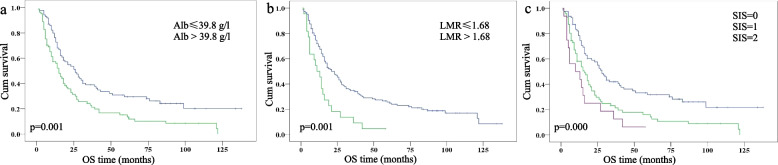
Kaplan—Meier analysis of overall survival according to (**a**) albumin levels (Alb), (**b**) lymphocyte-to-monocyte ratio (LMR), and (**c**) systemic inflammation score (SIS)

**Fig. 2:**
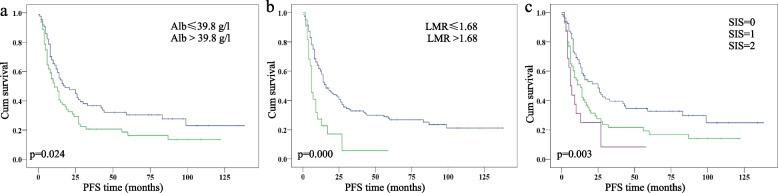
Kaplan—Meier analysis of progression-free survival according to (**a**) albumin levels (Alb), (**b**) lymphocyte-to-monocyte ratio (LMR), and (**c**) systemic inflammation score (SIS)

The optimal cut-off values for Alb and LMR were 39.8 g/L and 1.68, respectively. The Alb and LMR were divided into two groups, based on the cut-off value. As shown in Fig. 1a and b, a decreased Alb and LMR were both associated with shorter OS (*p* = 0.001 for both). Kaplan–Meier analysis indicated that the OS of the SIS = 0, SIS = 1 and SIS = 2 groups was 28.0 ± 2.9, 16.0 ± 2.8 and 10.0 ± 7.0 months, respectively (*p* = 0.000). SIS = 0 showed significantly longer OS than SIS = 1 and SIS = 2, respectively (*p* = 0.001 for both, Fig. 1c). The 3-year survival rates in the SIS = 0, SIS = 1 and SIS = 2 groups were 42% ± 16%, 23% ± 10%, and 19% ± 18%, respectively. Similar differences were also observed in PFS (Fig. 2).

### Univariate and multivariate Cox regression analyses for OS and PFS

The results from the univariate analysis indicated that NLR, PLR, SII, Alb and LMR as continuous variables were prognostic factors of OS as well as tumour location, T stage, M stage, TNM stage, radiotherapy technique, and concurrent chemotherapy (Table 3). Subsequent multivariate analysis identified T stage, M stage, concurrent chemotherapy, PLR and Alb as independent prognostic factors for OS. On the other hand, tumour location, M stage, radiotherapy techniques, early tumour response and PLR were independent prognostic factors for PFS (Table 4).

**Table 3:** Univariate and multivariate analyses of OS in the models with or without SIS factor

Prognostic factors	Univariate analysis		Multivariate analysis^a^		Multivariate analysis^b^	
	***P***	**HR(95%CI)**	***P***	**HR(95%CI)**	***P***	**HR(95%CI)**
Age (≤ 70 years vs. > 70 years)	0.103	1.34(0.94–1.89)				
Gender (male vs. female)	0.598	0.90(0.62–1.32)				
KPS (< 80 vs. ≥ 80)	0.257	0.70(0.38–1.30)				
BMI(< 18.5 kg/m^2^ vs. ≥ 18.5 kg/m^2^)	0.900	0.97(0.58–1.63)				
Smoking status (no vs. yes)	0.206	0.80(0.57–1.13)				
Tumour location (cervical + upper thoracic vs. middle and low thoracic)	**0.003**	1.73(1.20–2.49)				
T stage (T1-2 vs. T3-4)	**0.027**	1.76(1.07–2.91)	**0.019**	1.83(1.10–3.04)	**0.014**	1.88(1.14–3.12)
N stage (N0 vs. N1)	0.362	1.22(0.80–1.86)				
M stage (M0 vs. M1)	**0.008**	1.62(1.13–2.31)	**0.006**	1.70(1.16–2.49)	**0.018**	1.58(1.08–2.32)
Tumour TNM stage (I + II vs. III + IV)	**0.017**	1.74(1.10–2.73)				
Radiotherapy techniques (2D-RT vs. 3D-RT/IMRT)	**0.050**	0.67(0.45–1.00)				
Concurrent chemotherapy (0 vs. 1 vs. 2)	**0.002**	0.72(0.59–0.89)	**0.007**	0.74(0.59–0.92)	**0.006**	0.73(0.59–0.92)
Tumour early response(CR/PR vs. SD/PD)	0.156	1.31(0.90–1.90)				
Family history of cancer (no vs. yes)	0.820	1.06(0.66–1.69)				
NLR^c^	**0.003**	1.12(1.04–1.20)				
PLR^c^	**0.001**	1.00(1.00–1.00)	**0.013**	1.00(1.00–1.00)		
SII^c^	**0.000**	1.00(1.00–1.00)				
Alb^c^	**0.003**	0.94(0.91–0.98)	**0.025**	0.95(0.91–0.99)		
LMR^c^	**0.012**	0.89(0.82–0.98)				
SIS (0 vs. 1 vs. 2)	**0.000**	1.70(1.32–2.19)			**0.003**	1.50(1.15–1.96)

The *P* value in bold indicated that statistically significant

*Abbreviations*: *OS* Overall survival, *KPS* Karnofsky performance status, *BMI* Body mass index, *IMRT* Intensity modulated radiation therapy, *CR* Complete response, *PR* Partial response, *SD* Stable disease, *PD* Progressive disease, *Alb* Serum albumin, *LMR* Lymphocyte-to-monocyte ratio, *SIS* Systemic inflammation score

^a^Adjustment for tumour location, T stage, M stage, tumour TNM stage, radiotherapy techniques, concurrent chemotherapy, NLR, PLR,Alb and LMR

^b^Adjustment for tumour location, T stage, M stage, tumour TNM stage, radiotherapy techniques, concurrent chemotherapy and SIS

^c^Analysed as a continuous variable

**Table 4:** Univariate and multivariate analyses of PFS in the models with or without SIS factor

Prognostic factors	Univariate analysis		Multivariate analysis^a^		Multivariate analysis^b^	
	***P***	**HR(95%CI)**	***P***	**HR(95%CI)**	***P***	**HR(95%CI)**
Age (< 70 years vs. ≥ 70 years)	0.285	1.23(0.84–1.80)				
Gender (male vs. female)	0.209	0.77(0.51–1.16)				
KPS (< 80 vs. ≥ 80)	0.814	0.92(0.45–1.88)				
BMI(< 18.5 kg/m^2^ vs. ≥ 18.5 kg/m^2^)	0.804	1.07(0.63–1.82)				
Smoking status (no vs. yes)	**0.024**	0.65(0.45–0.94)				
Tumour location (cervical + upper thoracic vs. middle and low thoracic)	**0.000**	2.08(1.39–3.09)	**0.011**	1.75(1.14–2.70)	**0.028**	1.63(1.05–2.53)
T stage (T1-2 vs. T3-4)	0.064	1.67(0.97–2.89)				
N stage (N0 vs. N1)	0.448	1.19(0.76–1.84)				
M stage (M0 vs. M1)	**0.000**	1.97(1.36–2.85)	**0.010**	1.67(1.13–2.48)	**0.022**	1.60(1.07–2.38)
Tumour TNM stage (I + II vs. III + IV)	**0.016**	1.84(1.12–3.01)				
Radiotherapy techniques (2D-RT vs. 3D-RT/IMRT)	**0.007**	0.57(0.38–0.86)	**0.049**	0.66(0.44–1.00)	**0.038**	0.65(0.43–0.98)
Concurrent chemotherapy (0 vs. 1 vs. 2)	**0.036**	0.79(0.64–0.98)				
Tumour early response(CR/PR vs. SD/PD)	**0.035**	1.52(1.03–2.24)	**0.010**	1.70(1.14–2.53)	**0.016**	1.63(1.10–2.43)
Family history of cancer (no vs. yes)	0.284	1.30(0.81–2.08)				
NLR^c^	**0.018**	1.10(1.02–1.19)				
PLR^c^	**0.006**	1.00(1.00–1.00)	**0.048**	1.00(1.00–1.00)		
SII^c^	**0.013**	1.00(1.00–1.00)				
Alb^c^	**0.018**	0.95(0.91–0.99)				
LMR^c^	**0.002**	0.86(0.78–0.95)				
SIS (0 vs. 1 vs. 2)	**0.001**	1.58(1.21–2.08)			**0.017**	1.40(1.06–1.88)

The *P* value in bold indicated that statistically significant

*Abbreviations*: *PFS* Progression free survival, *KPS* Karnofsky performance status, *BMI* Body mass index, *IMRT* Intensity modulated radiation therapy, *CR* Complete response, *PR* Partial response, *SD* Stable disease, *PD* Progressive disease, *Alb* Serum albumin, *LMR* Lymphocyte-to-monocyte ratio, *SIS* Systemic inflammation score

^a^Adjustment for smoking status, tumour location, M stage, tumour TNM stage, radiotherapy techniques, concurrent chemotherapy, tumour early response, NLR, PLR,Alb and LMR

^b^Adjustment for smoking status, tumour location, M stage, tumour TNM stage, radiotherapy techniques, concurrent chemotherapy, tumour early response and SIS

^c^Analysed as a continuous variable

SIS was based on the combination of serum albumin and LMR, thus we analysed the novel prognostic factor of SIS for OS and PFS using univariate and multivariate Cox regression analyses. The results of multivariate analysis identified SIS as an independent prognostic factor for OS (HR = 1.50 (95% CI, 1.15–1.96); *p* = 0.003; Table 3) and PFS (HR = 1.40 (95% CI, 1.06–1.88); *p* = 0.017; Table 4). The other independent prognostic factors were T stage, M stage, concurrent chemotherapy for OS and tumour location, M stage, radiotherapy techniques, and early tumour response for PFS.

### Predictive nomogram for OS

**Fig. 3:**
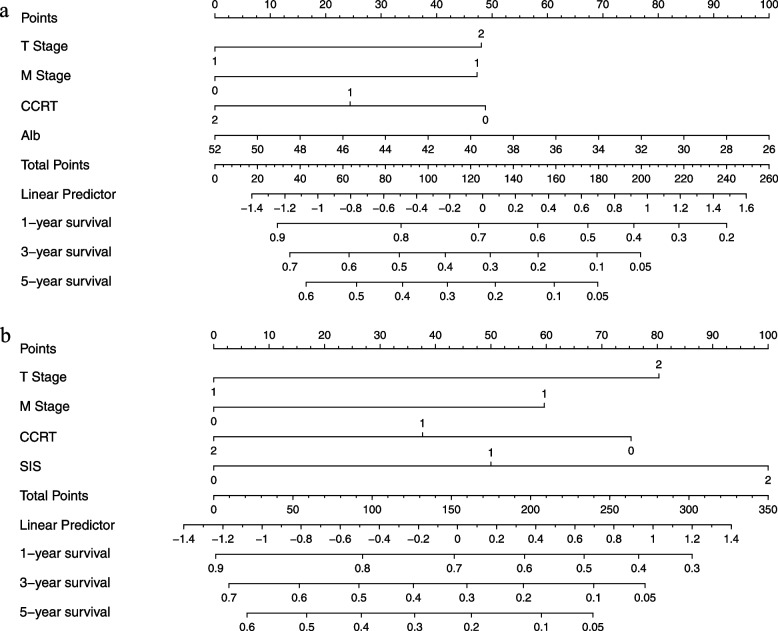
Nomograms for predicting 1-, 3- and 5-year overall survival of elderly oesophageal patients based on multivariate analysis in the model (**a**) without SIS factor, (**b**) with SIS factor

We constructed two nomograms based on the results of multivariate analysis. Nomogram 1 integrated the proven independent prognostic factors consisting of T stage, M stage, concurrent chemotherapy and continuous variable Alb (Fig. 3a). Nomogram 2 contained the novel predictor factor SIS and T stage, M stage and concurrent chemotherapy (Fig. 3b). In both nomograms, a higher total point indicates a worse prognosis. The C-index of Nomogram 1 and Nomogram 2 was 0.661 and 0.677, respectively. SIS showed a better predictive ability for OS than the continuous variable Alb.

### SIS stratified patient prognosis in different therapeutic regimens

**Fig. 4:**
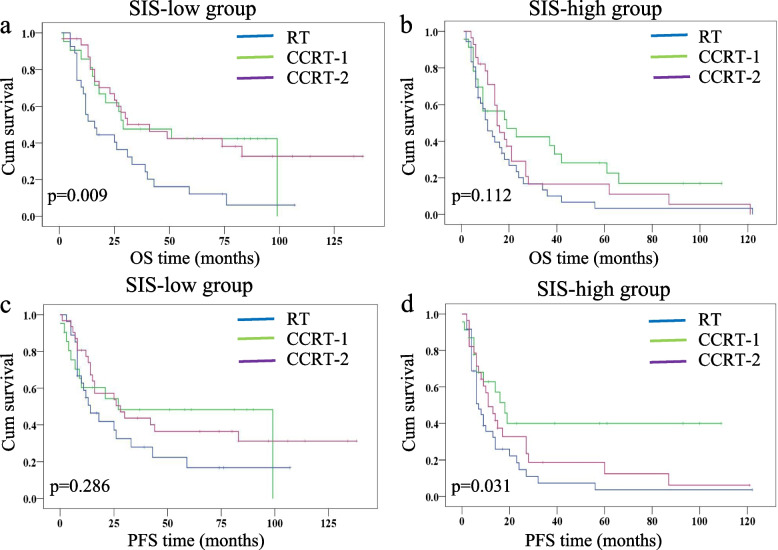
Kaplan—Meier analysis for OS and PFS according to treatment strategies in different SIS groups. **a** OS in the SIS-low group, **b** OS in the SIS-high group, **c** PFS in the SIS-low group, **d** PFS in the SIS-high group

A total of 79 (47.6%), 71 (42.8%) and 16 (9.6%) patients were allocated to SIS = 0, SIS = 1 and SIS = 2 groups, respectively. There were few patients in the SIS = 2 group, and the OS was similar in the SIS = 1 and SIS = 2 groups (Fig. 1c). Thus, in this section, we divided SIS into two groups: SIS-low (SIS = 0) and SIS-high (SIS = 1 and SIS = 2). Kaplan—Meier analysis was carried out to explore the influence of SIS on the systemic treatment response of elderly ESCC patients. In the SIS-low group, the median OS was 16.0 ± 4.3 months, 29.0 ± 17.7 months, and 41.0 ± 13.4 months for patients who received concurrent chemoradiotherapy with radiotherapy alone (RT), patients who received concurrent chemoradiotherapy with a single agent (CCRT-1) and patients who received concurrent chemoradiotherapy with two agents (CCRT-2), respectively. In this population, patients who were treated with CCRT-1 and CCRT-2 had better OS than patients treated with RT (*p* = 0.028 and *p* = 0.005, respectively) (Fig. 4a). The PFS in the SIS-low subgroups was not significantly different (14.0 ± 4.1 months for patients with RT; 27.0 ± 17.1 months for patients with CCRT-1 and 28.0 ± 9.5 months for patients with CCRT-2, *p* = 0.286) (Fig. 4c). In the SIS-high group, patients treated with CCRT-1 had a longer OS than patients who received RT (19.0 ± 10.4 versus 11.0 ± 2.2 months, *p* = 0.047), but a comparable OS to patients who received CCRT-2 (19.0 ± 10.4 versus 15.0 ± 1.3 months, *p* = 0.422, Fig. 4b). Similar differences were also observed in PFS (7.0 ± 1.4 months for patients with RT; 18.0 ± 3.3 months for patients with CCRT-1 and 11.0 ± 2.5 months for patients with CCRT-2; Fig. 4d). It is worth mentioning that the 3-year OS in patients treated with CCRT-1 and patients treated with CCRT-2 was 42% ± 11% and 15% ± 6%, respectively. The difference was significantly different compared with the log-rank test (*p* = 0.039).

### Time-dependent ROC Curve Analysis

**Fig. 5:**
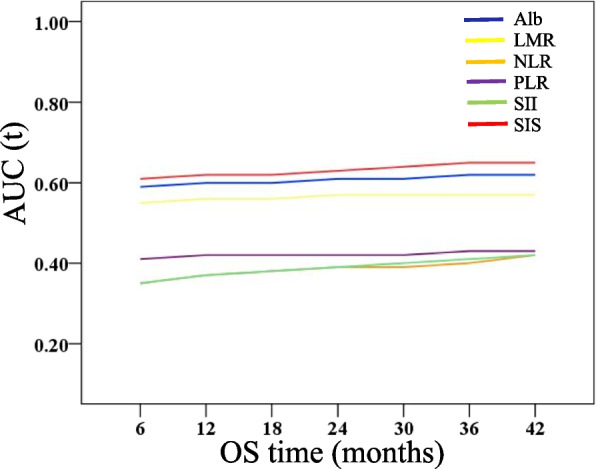
Time-dependent receiver operating characteristic curves of Alb, LMR, NLR, PLR, SII and SIS for the prediction of overall survival

We compared the prognostic impact of the SIS with other prognostic factors (Alb, LMR, NLR, PLR, and SII). Analysis of the time-dependent receiver operating characteristic curves for predicting OS using Hiplot software (https://hiplot.com.cn/) showed that the SIS exhibited a greatest AUC value than the other inflammatory indicators at each survival endpoint (Fig. 5).

## Discussion

Surgery is the backbone of curative-intent treatment for locally advanced resectable oesophageal cancer, although definitive chemoradiotherapy is also a recommended option [20]. However, most elderly patients are referred to definitive chemoradiotherapy because they have multiple comorbidities, poor nutritional status and poor surgical tolerance, which are often associated with a worse long-term and short-term prognosis. Notably, in our study, 34 patients (21.4%) with stage I-II disease were treated with definitive chemoradiotherapy but not surgery for various reasons. 6 patients rejected surgery, 5 patients had tumours in the cervical segment, 5 patients were aged older than 80 and rejected surgery, 5 patients had tumours longer than 7 cm according to gastroscopy, 3 patients had poorly controlled hypertension and diabetes, 3 patients had poor BMI, 1 patient had heart disease, and 6 patients had other unknown reasons. Therefore, the identification of novel prognostic indicators to predict the prognosis of elderly oesophageal cancer patients receiving radiotherapy or chemoradiotherapy is necessary.

The systemic inflammation score (SIS) is a novel marker consisting of serum albumin (Alb) level and lymphocyte-to-monocyte ratio (LMR). Several studies have demonstrated the predictive value of SIS in patients undergoing curative resection for various types of cancers [16–19], but few have examined the predictive role of SIS in elderly oesophageal cancer patients treated with radiotherapy. In the current study, we enrolled elderly patients as the research population and assessed the clinical influence and survival predictive value of SIS in elderly ESCC patients who did not undergo surgery. We demonstrated that in multivariate analysis of the model without the SIS factor, Alb and PLR were independent prognostic factors for OS and PFS, respectively. The multivariate analysis of the model with the SIS factor revealed that the SIS was a significant independent biomarker for predicting OS and PFS. Moreover, integrated or not integrated SIS factor in the prognostic nomogram showed that the C-index was improved to 0.677 when the SIS factor was incorporated. This meant that SIS had a better predictive ability for OS than Alb. Our previous research [3] found that concurrent chemoradiotherapy was an acceptable treatment for elderly patients with ESCC, but patients treated with CCRT-1 had comparable survival outcomes to patients treated with CCRT-2. The current study explored the predictive value of the SIS in stratifying prognosis in patients receiving CCRT-1 and CCRT-2. The results showed that the 3-year OS rate in patients treated with CCRT-1 was higher than that in patients treated with CCRT-2 in the SIS-high group (42% versus 15%, *p* = 0.039), which suggests that CCRT-1 is the better treatment choice for patients in the SIS-high group.

As an integrated indicator based on Alb and LMR, the mechanism of the prognostic value of SIS might be attributed to the function of the albumin, lymphocytes and monocytes. Alb is synthesized specifically in the liver and represents a malnutrition-inflammation status [21]. A decline in Alb levels implies poor outcomes in many types of cancer [22–24]; lymphocytes are immune cells that inhibit tumour development by enhancing the cancer immunosurveillance [25]. Monocytes can be recruited to carcinoma tissues and develop into tumour-infiltrating macrophages, which promote tumour growth [26, 27]. Therefore, a higher lymphocyte-to-monocyte ratio (LMR) might be associated with a good prognosis, which has been confirmed in previous studies [28–30]. In agreement with these facts, the predictive marker SIS was negatively associated with survival outcomes in our study. Notably, the predictive nomogram of OS indicated that the SIS had a better predictive ability for OS than Alb.

A link between cancer and inflammation was originally made by Virchow in 1863 [31]. Accumulating evidence suggests that cancer-related inflammation contributes to the progression of cancer by changing the tumoral microenvironment [8, 32]. In addition, cancer-related inflammation also induced changes in the peripheral blood, including the total of neutrophil, lymphocyte, platelet levels, and monocyte levels. Several combinations such as LMR [33, 34], NLR [35], PLR [36] and SII [37, 38] were all proven to be associated with the clinical outcome of oesophageal cancer. However, the AUCs of NLR, PLR and SII were significantly lower than the AUC of SIS, meaning that SIS showed superior accuracy compared with other systemic inflammation indexes (Alb, LMR, NLR, PLR, and SII) for the prediction of OS in our study.

Limitations should be addressed as follows. We must note that this was a retrospective study, and our findings should be validated by a multicentre study with a larger sample size. On the other hand, the systemic inflammation indexes (Alb, LMR, NLR, PLR, SII and SIS) were peripheral blood-based biomarkers. However, a generally accepted thresholds for peripheral blood-based biomarkers do not exist. In the present study, the optimal cut-off values of those indexes were calculated by X-tile software.

## Conclusions

SIS is a simple, economic and convenient scoring system for predicting the prognosis of elderly ESCC patients receiving radiotherapy or chemoradiotherapy. SIS could stratify patient prognosis in different therapeutic regimens, helping the physicians make more appropriate treatment choices for patients. CCRT-1 may be the best treatment for SIS-high patients.

## Data Availability

All data generated or analysed during this study are included in this published article and its supplementary information files.

## Abbreviations

SIS: Systemic inflammation score
ESCC: Oesophageal squamous cell carcinoma
CCRT-1: Concurrent radiotherapy with a single agent
CCRT-2: Concurrent radiotherapy with two agents;
Alb: Serum albumin level
LMR: Lymphocyte-to-monocyte ratio
NLR: Neutrophil-to-lymphocyte ratio
PLR: Platelet-to-lymphocyte ratio
SII: Systemic immune-inflammatory index
KPS: Karnofsky performance status
BMI: Body mass index
PFS: Progression-free survival
OS: Overall survival
